# A Programmable Digital Microfluidic Assay for the Simultaneous Detection of Multiple Anti-Microbial Resistance Genes

**DOI:** 10.3390/mi8040111

**Published:** 2017-04-01

**Authors:** Sumit Kalsi, Samuel L. Sellars, Carrie Turner, J. Mark Sutton, Hywel Morgan

**Affiliations:** 1 Electronics and Computer Science, Institute for Life Sciences, University of Southampton, Southampton SO17 1BJ, UK; sk24g09@soton.ac.uk (S.K.); sls1e14@soton.ac.uk (S.L.S.); 2 National Infections Service, Public Health England, Porton Down, Salisbury SP4 0JG, UK; Carrie.Turner@phe.gov.uk (C.T.); Mark.Sutton@phe.gov.uk (J.M.S.)

**Keywords:** isothermal amplification, RPA, digital microfluidics (DMF), antimicrobial resistance (AMR)

## Abstract

The rapid emergence of antimicrobial resistant bacteria requires the development of new diagnostic tests. Nucleic acid-based assays determine antimicrobial susceptibility by detecting genes that encode for the resistance. In this study, we demonstrate rapid and simultaneous detection of three genes that confer resistance in bacteria to extended spectrum β-lactam and carbapenem antibiotics; CTX-M-15, KPC and NDM-1. The assay uses isothermal DNA amplification (recombinase polymerase amplification, RPA) implemented on a programmable digital microfluidics (DMF) platform. Automated dispensing protocols are used to simultaneously manipulate 45 droplets of nL volume containing sample DNA, reagents, and controls. The droplets are processed and mixed under electronic control on the DMF devices with positive amplification measured by fluorescence. The assay on these devices is significantly improved with a Time to Positivity (TTP) half that of the benchtop assay.

## 1. Introduction

The emergence of antibiotic resistant bacteria is considered one of the greatest threats to human health as infections are becoming increasingly common [1]. The spread of resistance mechanisms has led to the continuous evolution of the β-lactamase enzymes against β-lactam antibiotics [2]. The production of extended spectrum β-lactamases (ESBLs) in Gram-negative bacteria is an example of a spreading problem fuelled by exposure of bacteria to cephalosporin antibiotics. Amongst the class A enzymes, the most common ESBL are those of the CTX-M family [3]. This gene has spread world-wide and is now found across at least 26 bacterial species [4,5]. Of the CTX-M family, CTX-M-15, encoded by *bla*_CTX-M-15_, is currently the most prevalent within the UK, whilst CTX-M-14 is more common in other parts of the world. Given the widespread emergence of ESBLs, carbapenems have been considered as the last resort antibiotic for the treatment of a range of multidrug resistant bacterial infections [6,7,8]. However, carbapenem resistance is also emerging, dramatically limiting treatment options. There are several carbapenemase enzymes, including the *Klebsiella pneumoniae* carbapenemase (KPC) and New Delhi metallo-β-lactamase (NDM). KPC is one of the most effective carbapenemases that can inactivate all basic β-lactams, cephalosporins, and carbapenems, but is partially inhibited by inhibitors such as clavulanic acid and tazobactam [6]. Spread of KPC has been driven in part by highly successful, multidrug-resistant strains of *K. pneumoniae*, although the gene is by no means limited to this pathogen. Conversely, NDM spread has been driven by the dissemination of plasmids, carrying the *bla*_NDM-1_ gene alongside a number of other antibiotic resistance markers [9]. This dissemination is of concern for public health worldwide, given the occurrence of the gene in unrelated species and acquisition by *E. coli*, which is one of the main community-acquired human pathogen. As carbapenems are the last resort for a number of multidrug resistant Gram-negative bacteria [7], a major cause of nosocomial and community bacterial infections, new methods are needed to maintain and extend the useful working life of this class of antibiotics. Current tests to determine whether bacteria are killed by antibiotics are based on the standard culture of bacteria in the presence of an antibiotic. These indicate susceptibility to different antibiotic classes and can be used to direct therapy, but are slow and take up to 72 h. In the absence of rapid results, treatment will often use broad-spectrum antibiotics on an empirical basis, which may contribute to increased drug resistance. The emergence of multi-drug-resistant (MDR) strains of bacteria is increasing the need for rapid determination of antimicrobial susceptibility [10], and nearly 200,000 people die every year from MDR tuberculosis (TB) alone [11]. Therefore, there is a clear need to develop rapid and sensitive tests to determine antibiotic resistance, both as part of the diagnosis and management of infection and to provide a rational basis for the appropriate prescription of antibiotics. 

A number of groups are pursuing the development of miniaturised methods for fast analysis of microorganism susceptibility to antibiotics. Examples include Boedicker et al. who confined individual bacteria into nL droplets to determine susceptibility of MRSA to several antibiotics [12]. Choi et al. demonstrated how imaging changes in bacterial morphology over time could be used as a rapid susceptibility assay [13]. Cira et al. described a portable microfluidic technology to determine the minimum inhibitory concentration (MIC) of antibiotics against bacteria using colorimetric readout [14]. Schoepp et al. determined susceptibility of Urinary tract infection (UTI) clinical isolates using digital PCR [15]. Interested readers are referred to reviews by O’Kelley [16] and by Pulido et al. [17].

In an alternative approach, we are developing approaches based on nucleic acid (NA) analysis as a rapid and sensitive method to directly identify the genes that confer resistance. It is particularly useful for slow growing or non-culturable microorganisms and for the detection of point mutations [18]. Polymerase chain reaction (PCR) is a well-known NA based amplification assay. It is generally performed in centralised labs with expert users and specialized equipment, although there are several examples of portable systems that can be used in the field. Multiplexed PCR assays for simultaneous detection of resistance genes have also been developed [19,20,21]; however, although PCR is the gold standard, the need for accurate temperature cycling makes the final device more complex. Therefore, isothermal DNA amplification methods that do not require thermal cycling are gaining popularity. One such technique is recombinase polymerase amplification (RPA), which is a simple and robust method of amplifying DNA [22,23]. It works with a wide range of sample matrices and requires only two primers. RPA uses recombinase enzymes for primer annealing and unwinding DNA. Real-time amplification of product can be monitored using a fluorescent readout using *exo* probes, a complimentary oligonucleotide that has a tetrahydrofuran (THF) moiety located between a fluorophore and a quencher. Exonuclease III in the reaction mix cleaves the THF once the probe has bound to the target sequence emitting fluorescence.

There is growing literature describing the development of isothermal amplification techniques for nucleic acid based rapid diagnostics. However, there are very few reports of multiplexed detection of different target genes on the same device. Notable examples include Czilwik et al. who developed a centrifugal microfluidic platform “LabDisk” and demonstrated multiplex PCR to identify four different microorganisms (*Staphylococcus warneri*, *Streptococcus agalactiae*, *Escherichia coli*, and *Haemophilus influenza*) in a small volume serum sample [24]. They used nested PCR and amplified DNA from each organism from a 200 μL serum sample and 20 μL PCR reaction volume chamber. Choi et al. also used a centrifugal platform to demonstrate a triplex isothermal RPA assay for identification of three different organisms in contaminated milk, using a 2.6 μL amplification volume [25]. The LoD of the system was four cells. In an alternative approach, Kersting et al. designed a simple chip capable of simultaneous multiplex isothermal amplification of DNA using RPA on a solid surface. The sample was continuously pumped across the surface of an 8.3 μL reaction chamber, and they were able to amplify specific target sequences from three pathogens *Neisseria gonorrhoeae*, *Salmonella enterica*, and methicillin-resistant *Staphylococcus aureus* (MRSA) using genomic DNA [26]. They demonstrated detection in 20 min and a LoD of extracted DNA from between 10 and 100 cfu. Crannell et al. demonstrated a triplex assay using RPA to detect DNA from the protozoa *Giardia*, *Cryptosporidium*, and *Entamoeba* using a lateral flow readout with limits of detection in the hundreds of copies [27]. Recently, a simple microfluidic device was demonstrated by Renner et al. that identifies ESKAPE bacterial pathogens using an RPA assay [28]. They utilized a vacuum degassed PDMS cartridge with 16 reaction chambers that contained lyophilized RPA assays. A portable electronic reader and end-point fluorescence detection was used to identify the pathogen.

Digital microfluidic (DMF) technology provides an alternative method for performing fully programmable automated microfluidic assays using nL volume liquid droplets. DMF exploits the phenomenon of electrowetting-on-dielectric (EWOD) to move liquids [29]. Droplets of liquid can be manipulated in two dimensions on the surface of an array of microelectrodes. Droplets with a wide range of volumes can be manipulated, and processes such as dispensing, splitting, mixing can all be accomplished using appropriate electrode patterns. Digital microfluidics has been used for many molecular assays [30,31], and, since it requires very small volumes, this often improves assay performance. Despite its flexibility, very few multiplexed assays have been demonstrated on DMF devices. Hua et al. demonstrated a two-plex PCR by cycling droplets repeatedly in a loop between two zones of different temperature. They showed that DNA from MRSA and *Streptococcus pneumoniae* could be detected in real time with single copy sensitivity [32]. More recently, Prakash et al. demonstrated a chip for the detection of influenza A virus and influenza B virus in clinical samples using RT-PCR [33].

Traditional “passive” DMF devices have a relatively small number of fixed hard-wired electrodes and the droplet manipulation operations (e.g., movement, shape) are limited to these electrodes patterned on the device. A new generation of DMF devices controlled by thin film transistor (TFT) electronics has recently been developed [34,35]. These active matrix AM-EWOD systems have many thousands of individually addressable electrodes enabling the simultaneous and independent manipulation of numerous droplets in 2D. They provide a high level of flexibility in defining droplet size and shape. The AM-EWOD devices also incorporate electrical droplet sensing functions to monitor the position and size of droplets in almost real time. These programmable DMF devices are therefore ideally suited for complex analytical processes providing a highly flexible platform capable of performing assays with nanolitre volume droplets.

Previously, we have demonstrated the enhancement in gene detection time and limit of detection as compared to benchtop RPA assays on the AM-EWOD platform [35]. In this paper, we further exploit the programmable capability of the active matrix DMF technology to amplify DNA extracted from antibiotic resistant bacteria in a triplex assay. All procedures for aliquoting, dispensing, and mixing samples were performed on the device. Specifically, the triplex assay identifies and can quantify three of the key genes responsible for antimicrobial resistance in Gram-negative microorganisms in a mixed sample droplet in a short time window. These genes are NDM-1, KPC, and CTX-M-15 that encode for carbapenemases and an ESBL respectively. The genes were identified using isothermal amplification methods and a fluorescent readout.

## 2. Methods

### 2.1. Digital Microfluidic (DMF) Device

**Figure 1:**
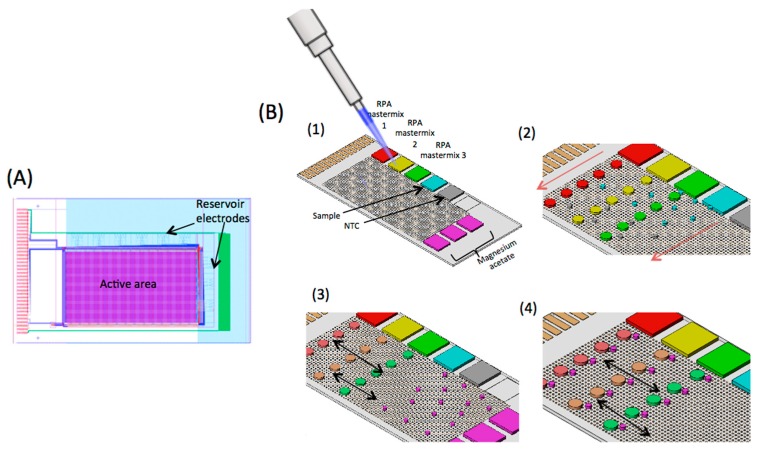
(**A**) Schematic representation of the TFT substrate of the AM-EWOD digital microfluidic platform. The active area is approximately 20 mm × 37 mm. It consists of 96 × 175 individually addressable circuit elements (or electrodes) within the active area. At the periphery of the device, there are 9 fluid input structures with large reservoir electrodes. Each input structure has a volume of 2.2 μL. (**B**) (**1**) Reagents required for the RPA assay are manually loaded onto the reservoir electrodes. These are RPA mastermix (specific to the gene of interest), sample, no template control (NTC), and magnesium acetate (to initiate the reaction). RPA mastermix 1, 2, and 3 contains primers and fluorescence probes for KPC, NDM-1 and CTX-M-15 genes respectively. (**2**) Daughter droplets are dispensed from the reservoirs. Sample and NTC droplets have a volume of 45 nL while RPA mastermix has a 180 nL volume. Red arrow represents the direction of dispense of the daughter droplets (see Video S1 in Supplementary Materials). (**3**) RPA mastermix droplets are mixed with sample or NTC droplets (volumetric ratio 4:1). Magnesium acetate droplets are also dispensed from the three reservoirs along the short edge of the TFT substrate. Black arrow represents the direction of droplet movement to facilitate mixing of RPA mastermix droplets with sample/NTC. (**4**) Magnesium acetate droplets are merged with the droplets containing DNA/NTC and RPA mastermix to initiate the reaction. The resulting droplets are moved on the active matrix (main array) of the TFT substrate (in the direction of black arrows) to thoroughly mix the contents of the reaction droplet. Following this step, the devices were heated to 39 °C and fluorescence recorded in real time. Illustration not to scale. For a video of the device in operation see Video S1.

The TFT substrate or backplane of the AM-EWOD DMF device is shown in Figure 1A and has been described in detail elsewhere [34,35]. It comprises a main array of 96 × 175 individually addressable circuit elements or pixels, each of 200 μm × 200 μm. The uppermost layer of the TFT substrate is indium tin oxide (ITO) electrode, to which a voltage is applied. Additional dielectric and hydrophobic layers are applied on these substrates. Adjacent electrodes are separated by a gap of 10 μm, giving a total active area of an array of 7.37 cm^2^. Immediately adjacent to the high-resolution array are nine fluid input structures that define a path for dispensing fluid onto the main array. Each of the input structure (3 mm × 9 mm) consists of seven electrodes which are formed in the same ITO layer as the main array and are controlled by the same TFT circuitry as the main array. Each pixel also contains a sensor for measuring the capacitance of the droplet, as described previously [34]. An image from the sensors shows the size and position of droplets on the array. Image processing techniques have been developed to measure the size of the droplets from the output sensor image, as described in [34]. The actuation pattern can be re-written 50 times per second and a sensor image obtained 30 times per second. The sensor thus facilitates real time feedback of droplet size and position. A temperature sensor is also integrated onto the TFT substrate to regulate the temperature of the built-in heater when used for DNA amplification chemistry. 

The complete DMF device consists of the TFT backplane and the transparent top ITO plate, both of which are coated with Cytop. The two glass substrates are separated by a spacer, typically 125 μm. The space between the two plates (top and bottom) is filled with dodecane. The thin film electronics layers up to and including ITO electrodes were fabricated using the Sharp CG Silicon TFT manufacturing process. An insulation layer of Al_2_O_3_ (300 nm) was deposited by atomic layer deposition (ALD) over the electronics and a hydrophobic top layer formed by spin coating a thin layer (approximately 80 nm) of Cytop (Asahi Glass, Tokyo, Japan). This cytop coating was stripped and replaced for each new DNA assay. 

### 2.2. Control Electronics and Software

A printed circuit board (PCB) supplies the voltage and timing signals to drive the TFT electronics. Control firmware (VHDL) and application software (C#) were custom designed for automated control of droplets. A set of droplet operations including move, merge, split, dispense, and mix is implemented by software through a graphical user interface. Droplet operations are pre-programmed by the user and implemented with real time feedback from the sensor. Droplets can also be manipulated through ‘click and drag’ operations on the sensor image. 

### 2.3. Bacterial Culture and Plasmid Extraction

Plasmids were extracted from an overnight culture of control strains based on *E. coli* Top 10 using QIAprep Spin Miniprep Kit (Qiagen, Manchester, UK) using the manufacturer’s protocol. The bacteria strains contains the plasmid pACYC184 carrying the ESBL or carbapenemase genes together with 300 to 500 bp upstream DNA, on a BamHI and XbaI fragment. The eluted plasmid was quantified on a Qubit^®^ fluorometer using an assay reagent.

### 2.4. Benchtop RPA Assay

Primer sets within the *bla*_CTX-M-15_, *bla*_KPC_ and *bla*_NDM-1_ gene were designed and tested in multiple combinations. The sequences for the primers and probes used in the study are available on request from Public Health England. The commercial TwistAmp^®^ Exo kit (TwsitDx, Cambridge, UK) was used for real-time RPA assays, following the manufacturer’s instructions. Lyophilised RPA proteins were reconstituted with rehydration solution, forward and reverse primers, a fluorescent exo-probe and DNA samples containing plasmids with CTX-M-15, NDM-1, or KPC genes. In each 50 μL reaction, the final concentration of primers was 0.42 μM, and CY5 or Quasar 670^®^ labelled probe was 0.12 μM, respectively. A 5 μL aliquot of the plasmid extracted from bacteria was added to this mix. Each RPA reaction mix was transferred to the well of a black polystyrene 96-well plate. Amplification was initiated by adding magnesium acetate to a final concentration of 14 mM and stirring vigorously. The plate was transferred to a GloMax microplate reader (Promega, Southampton, UK) set to 39 °C, and the fluorescence was measured at 1 min intervals for 30 min. 

### 2.5. DMF RPA Assay

For real-time RPA assay on the DMF platform, master droplets of reagents and sample (each with approximate volumes of 2 μL) were loaded directly onto the reservoir electrodes (Figure 1B). Tween^®^ 20 (molecular biology grade, Sigma Aldrich, Irvine, UK) was added to all the reagents to a final concentration of 0.1% *v*/*v* to reduce the surface tension of the droplets. As shown in the figure, the first three reservoir electrodes were loaded with three different RPA reaction mixes, with primers and probes specific to KPC, NDM-1, and CTX-M-15 genes. The other reservoir electrodes were loaded with sample (plasmid), nuclease free water (no template control, NTC) and magnesium acetate. The top substrate electrode was then clamped onto the TFT backplane, creating a sandwich structure with a well-defined cell gap (125 ± 1 μm) between the two glass substrates. The cell gap was filled with dodecane while the reservoir electrodes were actuated to pin down the droplets. Pre-programmed electrode actuation sequences were used to perform the assay. The sequence was designed so that droplets do not cross over each other during manipulation, thus circumventing any issues of cross contamination between reaction droplets. The protocol was as follows. First, the required number and volume of droplets were dispensed from the input reservoirs. The volume ratios are set to 4:1:1 for the RPA reaction mix, sample, and magnesium acetate, respectively. The RPA reaction mix (RPA proteins, rehydration solution, primers and probe specific to a given gene) was prepared so that the final component concentrations in the reaction droplet were identical to those in the 50 μL benchtop assay. 

The number of activated pixels on the array defines the size of each daughter droplet. The daughter droplets (5 for each RPA reaction mix, 15 magnesium acetate, 9 DNA droplets (triplicate), and 6 NTC droplets) were dispensed from the reservoir electrodes with the following volumes: RPA reaction mix = 180 nL (6 × 6 elements); DNA, NTC, and magnesium acetate = 45 nL (3 × 3 elements). After dispensing, the daughter droplets were moved to pre-determined regions on the array under direct software control (Figure 1B). Reagent and sample droplets were mixed using a programmed mixing sequence, which repeatedly shuttles the droplets back and forth to reduce the mixing time [35]. The video (see Video S1 in Supplementary Materials) shows the sequence of droplet dispensing and mixing, speeded up 10×. The dispensing of RPA reagents (green) was done at half the speed of the magnesium acetate (red) because the RPA is much more viscous. During mixing, the droplets had an approximately rectangular shape. The final reaction droplet volume was 270 nL. The RPA reaction droplets were first mixed with the DNA (or NTC) and then with magnesium acetate (Figure 1B). The array was then heated to 39 °C to initiate the RPA reaction.

### 2.6. Optical System and Data Analysis

**Figure 2:**
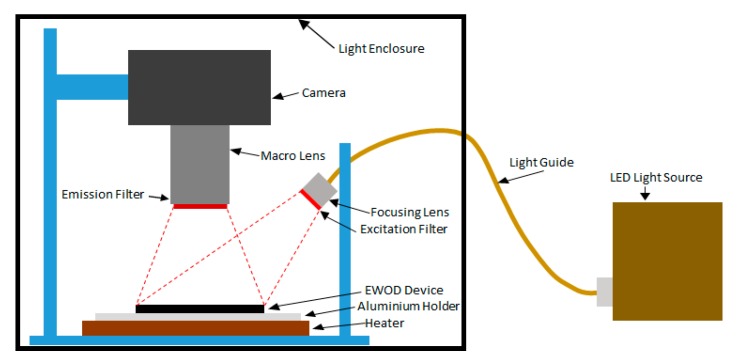
Diagram of the optical setup used for wide-field imaging of fluorescence from the RPA reaction droplets. LED light source provides polychromatic light, which passes through a collimator and is filtered by an excitation filter (590–650 nm). The fluorescence from the reaction droplets is recorded with a Canon 5D camera fitted with a macro lens. A bandpass filter (670–740 nm) is placed in the front of the camera to remove excitation light. Integration time is 2.5 s. Images recorded are analyzed with a custom MATLAB code to obtain real time fluorescence plots. The entire setup is placed in a light enclosure to eliminate stray light. Illustration not to scale.

A custom wide-field fluorescence detection system was made to image the entire device as shown in Figure 2. Light from an LED is directed on the chip by a focussing lens (100 mm, f2.8) and a band pass filter (590–650 nm, Semrock, Cambridgeshire, UK) to obtain the required wavelength for fluorophore excitation. The fluorescence emitted by the droplets was imaged by DSLR camera (Canon 5D, Mark III), fitted with a macro lens (55 mm focal length) and an emission filter (670–740 nm, Semrock). The droplets were exposed for 3 s and fluorescence recorded for a 2.5 s window during the exposure. An image was obtained every 30 s over 30 min using a custom software that controls the camera and light source. 

A custom MATLAB script was used to process and analyse the images to obtain fluorescence plots. First the positions of the droplets were selected manually before the images were batch processed. Droplet intensity data was recorded by taking the mean value of all pixels within the selected droplet region. The non-uniform light distribution produced by the source over the device area was determined individually for each image. This non-uniform distribution was calculated by multiplying the column vector of median values for each row in the image by the row vector of median values for each column in the image. Median values were used to avoid a significant impact on the background approximation from the droplets. The resulting image was then normalised. The original image was then divided by the approximated background to remove the non-uniformity. Figures S1 and S2 show an example of a processed and unprocessed image of fluorescent dye droplets on EWOD.

## 3. Results and Discussion

The sensitivity of the RPA assay for detection of the genes encoding CTX-M-15, NDM-1, and KPC was first assessed using the bench-top assay. A serial dilution of the three different plasmids was performed, with water as a negative control. Amplification curves (in triplicate) are shown in Figure S3. The assay was quantified through a measurement of the Time to Positivity (TTP), defined as the time at which the fluorescence signal crosses a point defined as three times the standard deviation (S.D.) of the negative controls. As expected, the TTP increased with decreasing plasmid concentration and a plot of TTP against logarithm of DNA concentration is shown in Figure S3D, where a log–linear relationship is seen, demonstrating the quantitative capability of the assay. As shown in the figure, all three genes can be detected in a similar time window. The benchtop assay can detect as few as 10 copies of CTX-M-15, NDM-1, and KPC within 25 min.

**Figure 3:**
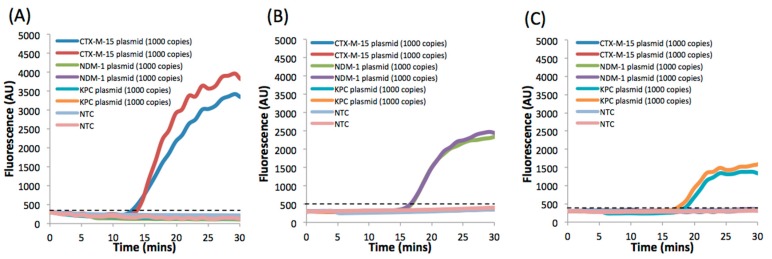
RPA amplification curve from benchtop reactions demonstrating the specificity of the triplex assay. RPA mastermix is prepared with primers and probes specific to the gene of interest. A sample containing all three plasmids CTX-M-15, NDM-1, and KPC is spiked in the reaction. Amplification curves are shown for RPA mastermix prepared with (**A**) CTX-M-15; (**B**) NDM-1; (**C**) KPC primers and probes. Dotted line represents threshold calculated as 3 × 1 S.D. of the NTC.

The specificity of the primers and probes for a given gene was also evaluated by amplifying samples without the specific genes present. Figure 3A shows amplification curves (in duplicates) for a sample containing three different genes (CTX-M-15, NDM-1, and KPC) in a reaction mixture containing primers and probes for only the CTX-M-15 gene. No amplification was observed for NDM-1 and KPC, while the fluorescence intensity from the sample containing plasmid with CTX-M-15 increased exponentially after a lag phase. Similarly, Figure 3B,C demonstrates the high specificity of the assay to the genes encoding for NDM-1 and KPC. The time to positivity (TTP) for a sample containing 1000 plasmid copies of CTX-M-15, NDM-1, and KPC was 13 ± 0.5, 16.7 ± 0.1, and 17.6 ± 0.8 min, respectively. 

**Figure 4:**
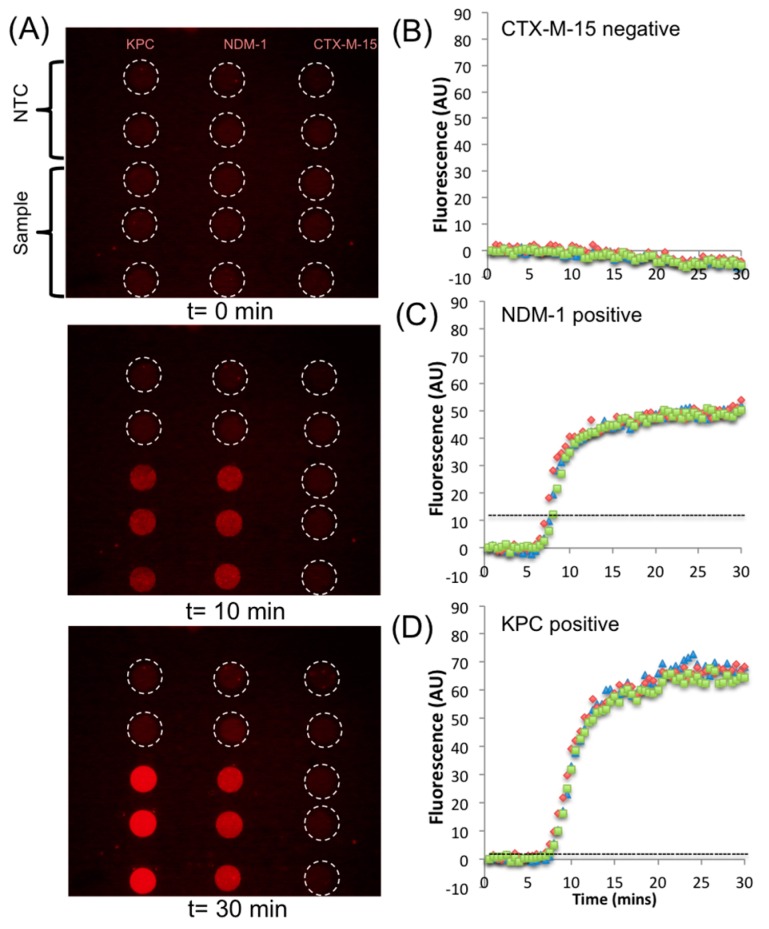
(**A**) Example fluorescence images of reaction droplets at time = 0, 10, and 30 min. This sample contains plasmid with KPC and NDM-1 (1000 copies of each). (**B**–**D**) DNA amplification curves (in triplicate) generated from the series of images (as shown in **A**) for CTX-M-15, NDM-1, and KPC. Dotted line is the threshold calculated as 3 × 1 S.D. of the NTC.

Using the droplet dispensing sequence outlined in Figure 1, the three different genes were also amplified and detected using RPA on the DMF platform. Figure 4A shows three images of the AM-EWOD, showing fluorescence from the droplets for the triple gene assay at *t* = 0, *t* = 10, and *t* = 30 min (after completion). There are five reaction droplets for each assay, corresponding to sample (in triplicate) and NTC (in duplicate), totalling 15 droplets representing 15 independent reactions. Each final droplet volume is approximately 230 nL.

As shown by the end-point fluorescence image, no DNA contamination occurred during droplet manipulation, as evidenced by the consistent lack of amplification in the no template control (NTC) reactions. The example reaction shown in Figure 4A used droplets with plasmids and genes encoding for NDM-1 and KPC; thus, the reaction showed positive for those genes and negative for CTX-M-15. Figure 4B–D shows the amplification curves for the droplet reactions in Figure 4A obtained from the fluorescent images. The TTP for NDM-1 and KPC for 1000 copies is 7.3 ± 0.3 and 7.4 ± 0.3 min, respectively, which is twice as fast as the bench top assay consistent with previous observations [28]. 

**Figure 5:**
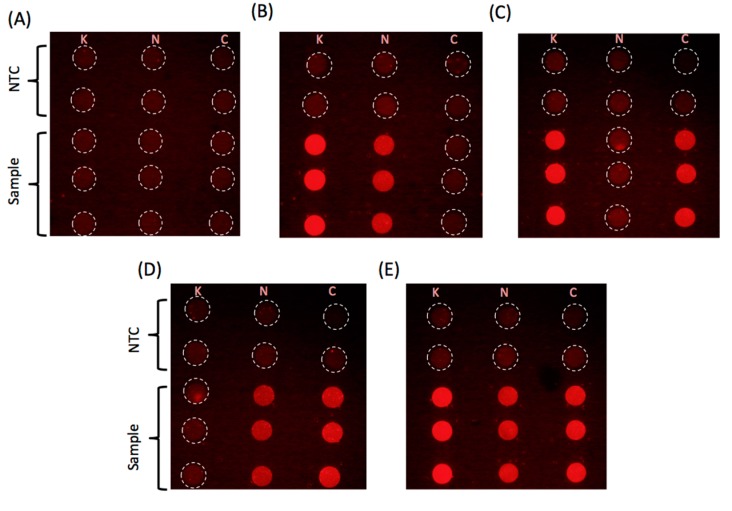
End point fluorescence images for reaction droplets at *t* = 30 min. Sample are as follows: (**A**) no plasmid; (**B**) plasmids with KPC and NDM-1; (**C**) plasmids with KPC and CTX-M-15; (**D**) plasmids with NDM-1 and CTX-M-15; (**E**) plasmids with CTX-M-15, KPC and NDM-1 (1000 copies each). Reaction was run in triplicate on the devices. The headers K, N, and C identify column with droplets that contain primers and probes for KPC, NDM-1, and CTX-M-15, respectively. See Figures S4 and S5 for DNA amplification curves corresponding to the images shown in B, C and D, E.

Finally, Figure 5 shows a series of images for samples with different combination of the three plasmids at 1000 copies per droplet. The images consistently indicate that no amplification occurs in the droplet with the missing gene demonstrating the excellent specificity of the assay. The amplification curves for the reactions extracted from the fluorescence intensity data can be seen in Figures S4 and S5.

## 4. Conclusions

In this work, we have demonstrated an automated multiple gene detection system using digital microfluidic AM-EWOD devices with isothermal RPA DNA amplification. The programmable device was used to implement a triplex assay to amplify the three most prominent genes conferring resistance to cephalosporins and carbapenems in Gram-negative bacteria. Sample DNA, RPA reagents (including gene-specific primers and probes), magnesium acetate, and NTC were all dispensed from the reservoir droplets. For each assay, five reaction droplets (3 positive and 2 NTC) per gene were generated and fluorescence was measured after mixing the daughter droplets dispensed from the reservoirs. This entire sequence was done in parallel for all 15 reaction droplets. The assay is specific and sensitive and can detect all genes with a LoD of 10 copies in approximately 25 min on benchtop. The assays on the device are twice as fast as the same assay on benchtop demonstrating increased speed and sensitivity. Future development of an integrated sample preparation unit will lead to a complete system that can provide a rapid, simple and sensitive DNA detection system for multidrug resistant pathogens.

## Supplementary Materials

The following are available online at www.mdpi.com/2072-666X/8/4/111/s1. Video S1: Sequence of droplet movement used to simultaneously detect three genes encoding using RPA assay on the DMF platform. Green droplets (RPA mastermix, DNA and water) were dispensed slower than the MgAc droplets. This is because the RPA mastermix is viscous and needs to be moved slower for reproducible generation of daughter volume. DNA and water droplets were also moved slower in order to synchronize their position on the array with RPA mastermix daughter droplets. Video has been speeded up 10×. Figure S1: Image showing droplets of fluorescent dye (Atto633) on the DMF platform. Brighter droplets have a concentration of 10^−7^ M, while the darker droplets have a concentration of 10^−8^ M. The pipetted volume of the large droplets is 1.5 µL, and the small droplets is 0.5 µL. Dotted box shows low fluorescence intensity in the small droplets on the left corner of the image as compared to the small droplets in the middle of the image, due to non-uniformity of the light. Figure S2: Processed image of fluorescent dye droplets shown in Figure S1. After image processing, the smaller droplets on the left corner (within dotted box) of the image shows similar fluorescence intensity as the other small droplets. Figure S3: Average of triplicate RPA reactions on bench top for sample containing (A) CTX-M-15, (B) NDM-1, and (C) KPC genes. Dotted line represents threshold calculated as 3 sigma of NTC. (D) Plot for Time To Positivity (TTP) against logarithm of the copies of plasmid present in the reaction. Figure S4: RPA amplification curve (in triplicates) plotted using the series of images shown in Figure 5B,C for samples with different genes. For the plots shown, the sample contained plasmid with genes encoding for (A) KPC and NDM-1, and (B) KPC and CTX-M-15. Dotted line represents threshold calculated as 3 sigma of NTC. Figure S5: RPA amplification curve (in triplicates) plotted using the series of images shown in Figure 5D,E for samples with different genes. For the plots shown, the sample contained plasmid with genes encoding for (A) NDM-1 and CTX-M-15, and (B) KPC, NDM-1, and CTX-M-15. Dotted line represents threshold calculated as 3 sigma of NTC.
