# Supplementary material for: A Programmable Digital Microfluidic Assay for the Simultaneous Detection of Multiple Anti-Microbial Resistance Genes
Source: Micromachines (Basel). 2017 Apr 1;8(4):111. doi: 10.3390/mi8040111 (PMC6189955; doi:10.3390/mi8040111)
Supplement: Supplementary file 1 [file micromachines-08-00111-s001.zip › micromachines-178046-supplementary.pdf]

Supplementary Materials: Programmable Digital Microfluidic Assay for Simultaneous Detection of Multiple Anti-Microbial Resistance Genes

Sumit Kalsi, Samuel L. Sellars, Carrie Turner, J. Mark Sutton and Hywel Morgan

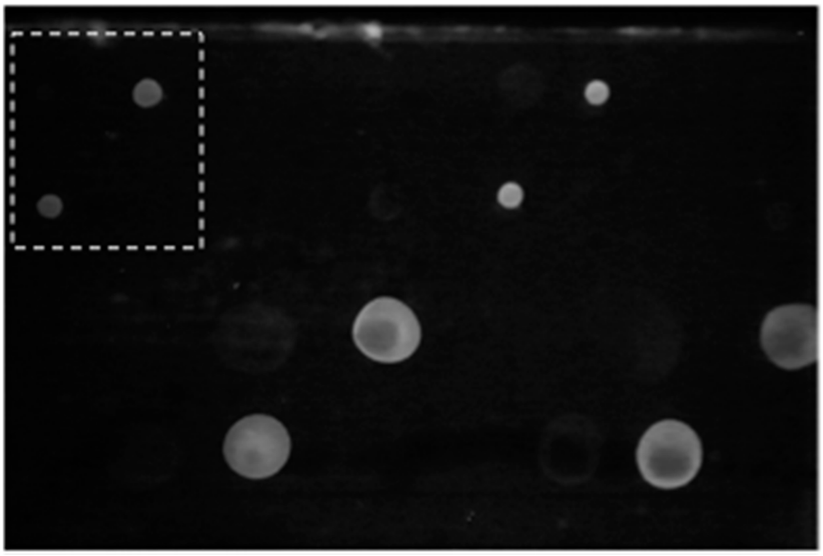

Figure S1. Image showing droplets of fluorescent dye (Atto633) on AM-EWOD platform. Brighter droplets have a concentration of 10^{-7} M, while the darker droplets have a concentration of 10^{-8} M. The pipetted volume of the large droplets is 1.5 μL, and the small droplets is 0.5 μL. Dotted box shows low fluorescence intensity in the small droplets on the left corner of the image as compared to the small droplets in the middle of the image, due to non-uniformity of the light.

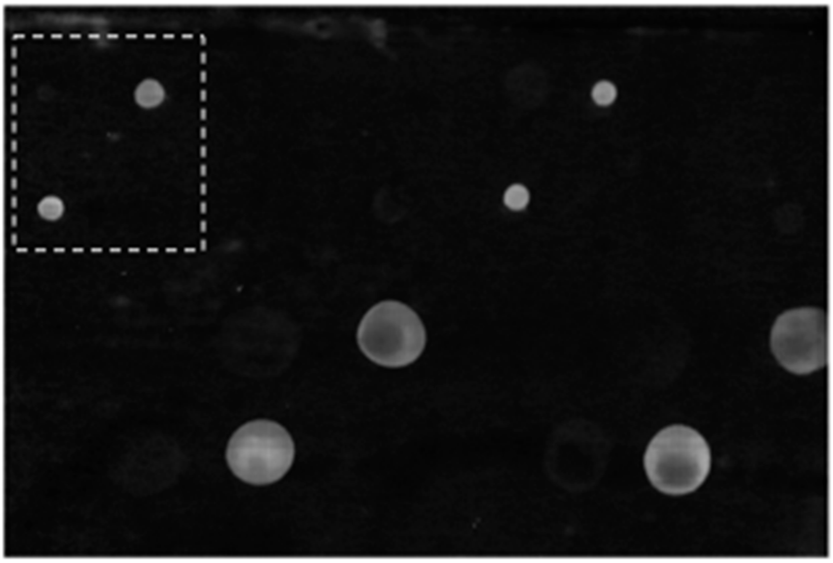

Figure S2. Processed image of fluorescent dye droplets shown in Figure S1. After image processing, the smaller droplets on the left corner (within dotted box) of the image shows similar fluorescence intensity as the other small droplets.

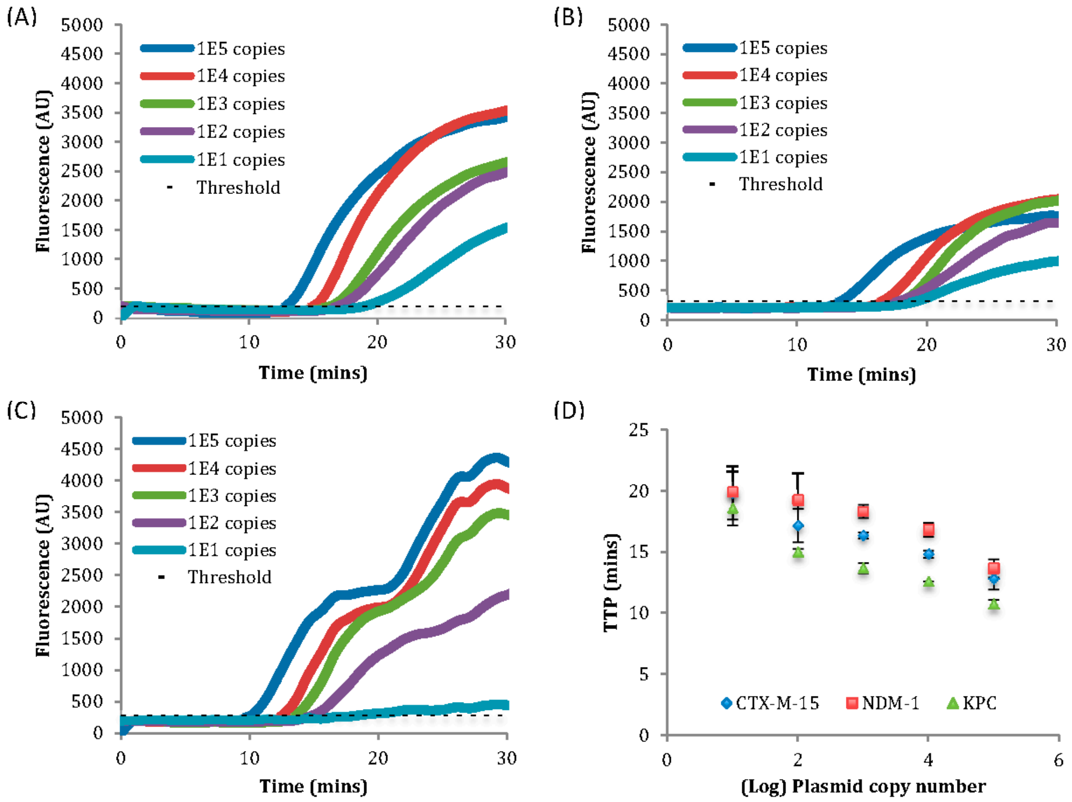

Figure S3. Average of triplicate RPA reactions on bench top for sample containing (**A**) CTX-M-15, (**B**) NDM-1, and (**C**) KPC genes. Dotted line represents threshold calculated as 3 sigma of NTC. (**D**) Plot for Time To Positivity (TTP) against logarithm of the copies of plasmid present in the reaction.

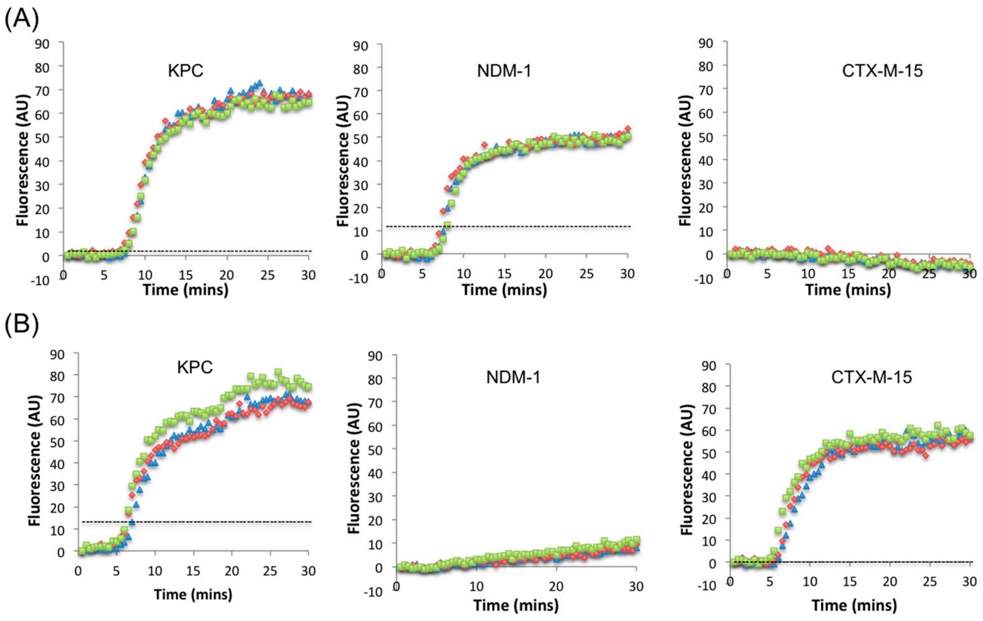

Figure S4. RPA amplification curve (in triplicates) plotted using the series of images shown in Figure 5B,C for samples with different genes. For the plots shown, the sample contained plasmid with genes encoding for (**A**) KPC and NDM-1, and (**B**) KPC and CTX-M-15. Dotted line represents threshold calculated as 3 sigma of NTC.

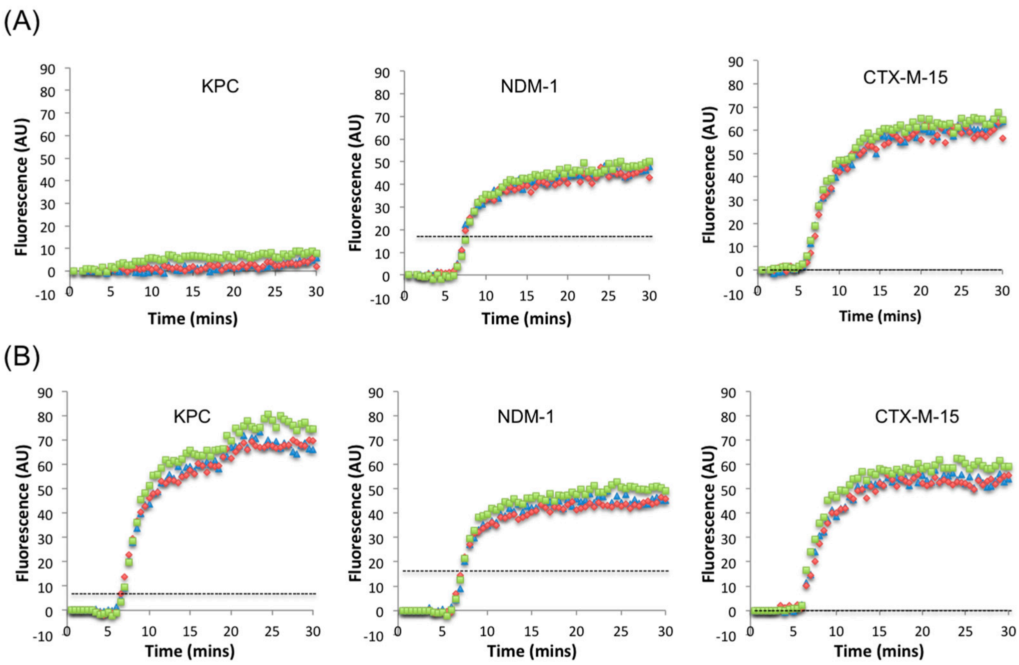

Figure S5. RPA amplification curve (in triplicates) plotted using the series of images shown in Figure 5D,E for samples with different genes. For the plots shown, the sample contained plasmid with genes encoding for (**A**) NDM-1 and CTX-M-15, and (**B**) KPC, NDM-1, and CTX-M-15. Dotted line represents threshold calculated as 3 sigma of NTC.
